# On the Use of Chloroform in the Treatment of Puerperal Insanity

**Published:** 1857-01-01

**Authors:** A. T. H. Waters

**Affiliations:** Lecturer on Anatomy and Physiology in the Liverpool Royal Infirmary School of Medicine— Surgeon to the Liverpool Dispensaries—Formerly Medical Attendant to the Liverpool Royal Lunatic Asylum


### Art. VIII.—ON THE USE OF CHLOROFORM IN THE TREATMENT OF PUERPERAL INSANITY.

BY A. T. H. WATERS,

*Lecturer on Anatomy and Physiology in the Liverpool Royal Infirmary School of Medicine—Surgeon to the Liverpool Dispensaries—Formerly Medical Attendant to the Liverpool Royal Lunatic Asylum.*

In a paper I recently read before the members of the Medical Society of Liverpool, I drew their attention to the great benefit to be derived from the use of anæthesia in the treatment of certain forms of puerperal mania. In consequence of the favourable opinions that have been expressed with reference to the paper, and as I believe the subject considered in it is one of much importance, I have been induced to revise the paper, and give it its present form.

The administration of chloroform in cases of mania is a subject that has already been under inquiry by those engaged in the treatment of insanity; but, whether from too much having been anticipated from it, from its having been indiscriminately used, or from some other cause, it seems to have fallen into disuse. I am not aware that it has ever been used extensively in such cases as I shall have to detail, or the principles on which alone I think it is likely to be beneficial, and which it is my intention to endeavour to lay down in the course of this paper.

It is in cases of puerperal mania that I have most extensively used the remedy, and although my experience of its use is not confined to such cases, it is of these I can speak with the greatest confidence.

It is not my intention to treat in a systematic manner of the disease under consideration, nor to review the various opinions that have been from time to time held with reference to its nature; nor shall I examine at any length into the value of the various modes of treatment which have at different times prevailed. My object is rather to endeavour to ascertain the cases in which the use of anæthesia is specially called for, and the nature of the symptoms it is calculated to control.

The statistics of insanity occurring in women show a considerable per-centage of cases of the puerperal form, that is say, of cases occurring after parturition, during, or immediately after, lactation. Of 3096 cases I have collected from various sources, 219 were cases of puerperal insanity, making about 7 per cent. This per-centage, however, is not a correct one, inasmuch as in the total number of cases women of all ages are included; whereas those past the period of child-bearing should be excluded, as no longer liable to the conditions in which puerperal insanity can occur. If such cases were omitted from the estimate, it is probable we should have a per-centage of about 10.

It seems to be a common opinion that cases of puerperal insanity very rarely, if ever, prove fatal; and it becomes an important consideration to correct this, which I believe to be an error likely to be attended with serious consequences. It is fortunately true that the majority of cases do recover, but the statistics of lunatic hospitals show a large per-centage of cases which remain incurable, a smaller, but by no means a satisfactory one, of cases which prove fatal.

The experience of practitioners with reference to recovery differs very materially, and I believe from this reason,—that some have observed cases in their private practice, and others in the practice of asylums; and inasmuch as those cases which are sent to asylums are generally of a far more serious character than those kept at home, it follows that the mortality in the one case will be greater than in the other.

The following table shows the rate of mortality, recovery, and incurability of 280 cases:—

| | | | | | |
|---|---|---|---|---|---|
| Of 92 | cases recorded by Esquirol, | 55 recovered, | 6 died, | 31 remained incurable. |
| 57 | ,, Dr. Burrows, | 35 | ,, 10 | 12 | ,, |
| 131 | ,, Dr. Webster, | 81 | ,, 6 | 44 | ,, |
| 280 | | 171 | 22 | 87 | |
| | | or, | or, | or, | |
| | | 61 per cent. | 7·85 p. ct. | 31 per cent. | |
| | | | or, | | |
| | | | 1 in 13. | | |

Dr. Copland states, from a series of cases he has collected, that about 4 in 5 recover, and 1 in 8 dies.

With the exception of a very few cases, in which the symptoms seem to be rather those of phrenitis than of mania, death does not take place at a very early period of the disease, but after the lapse of a considerable time, as in the cases mentioned by Dr. Webster, in which death took place at periods varying from 13 days to three months from the commencement of the attack.

The few statistics I have given will be sufficient for the purpose for which I have adduced them, viz.—to show the relative rates of mortality and curability of the disease: they indicate, at least as far as hospital cases are concerned, a rate of mortality which, although not high, is far from being of a satisfactory nature; and they tend to show that the disease does not kill at its onset, but after it has run a somewhat lengthened course.

It becomes necessary to examine into the causes of this rate of mortality, and to ascertain the nature of the symptoms which indicate danger, and which usually precede a fatal issue. On this subject I quote the following remarks from different authors. Dr. Copland says: "The chief danger in the disease, especially in the more pure or non-febrile form of it, arises from debility and exhaustion of nervous power; and this is the more to be dreaded when the disorder follows hæmorrhage or improper bleeding, when the pulse is very rapid, weak or small, or fluttering, and when there are great restlessness and long-continued want of sleep."

Dr. Reid, in an article in the Psychological Journal, remarks: "Exhaustion appears to be the principal source of danger; the want of sleep, intense excitement and monotonous self-fatigue, all combine to increase it; and it is often a matter of surprise to us, for what a length of time the human frame can withstand their effects. Should even the mental symptoms somewhat improve, yet if the insomnia still continue, with a quick pulse and other increasing symptoms of bodily debility, the termination of the case is to be looked for with apprehension."

Dr. Gooch, in his work on diseases of women, remarks "that his experience accords with that of Dr. Hunter, viz.—that there are two forms of puerperal mania; the one attended by fever, or at least the most important part of it, a rapid pulse; the other accompanied by a very moderate disturbance of the circulation: that the latter cases, which are by far the most numerous, recover, that the former generally die." Three cases reported by him "terminated fatally: all were attended by a very rapid pulse; some attended by a quick pulse recovered, but none of these were treated for paraphrenitis." He further states, speaking of favourable and unfavourable symptoms,—"Nights passed in sleep, a pulse slower and firmer, even though the mind continues disordered, promise safety to life; on the contrary, incessant sleeplessness, a quick, weak, fluttering pulse, and all the symptoms of increasing exhaustion, portend a fatal termination, even though the condition of mind may be apparently improved. In the cases which I have seen terminate fatally, the patient has died with symptoms of exhaustion, not with those of oppressed brain, excepting only one."

In the remarks I have quoted, I entirely concur. The danger of fatal issue does not depend upon any symptoms indicative of active mischief going on in the brain, or any other organ; the nervous excitement is not the result of inflammatory action in the brain, and in itself is secondary in point of danger to the exhaustion of nervous power and physical depression which supervene in consequence of the long-continued wakefulness, restlessness, and abstinence from food.

The refusal of food, which in severe cases of the disease is doggedly persisted in, is a matter for serious consideration, and it becomes, in fact, one of the most important symptoms to be dealt with. It is by no means uncommon for a patient under the influence of maniacal excitement to pass day after day, and night after night, even for weeks, in a state of continued restlessness and insomnia; and during this period there is obstinate refusal of food, and in some cases it becomes impossible to get the smallest quantity of nourishment into the system. Under these circumstances, it is not surprising that debility and physical exhaustion ensue, that the eye becomes haggard and the cheek sunken, that the heart becomes weak and rapid in its pulsations, and that ultimately the patient dies prostrate, whilst almost up to the period of dissolution the mental excitement continues. Should, however, death not result, the prolonged state of excitement and consequent debility will seriously impair the chances of perfect mental recovery, and increase the probability of termination in permanent mania.

The view taken above of the nature of the symptoms which indicate danger, seems to be borne out by the results of the *post-mortem* examinations of those cases that have terminated fatally. Esquirol remarks of the appearances in the cases he examined, "that, strictly speaking, they offer nothing in particular,—nothing, in fact, which enables us to recognise the material cause of the disease, or discover its seat."

The appearances described by observers in this country are almost entirely in accordance with the above. Dr. Burrows says, "The morbid appearances are not of a marked character. The pure cases of the malady present little beside anœmia of the brain and its membranes. Other morbid changes are simply coincident."

Dr. Webster has found "turgidity of blood-vessels of brain and membranes—effusion into fifth ventricle."

Dr. Gooch, in the *post-mortem* examinations he made, found "no disease of brain—blood-vessels of cranium generally empty."

From the consideration of the *post mortem* appearances in the fatal cases, and from the examination of the symptoms exhibited during life, it is scarcely possible to do otherwise than conclude that the disease is one of irritation rather than inflammation. This seems to be the opinion now generally entertained by those who have had most opportunities of observing the disease. The affection seems to be one in which the brain and nervous system generally are in a condition of great irritability; and this appears to be the result, more or less, of a state of exhaustion. The disease is one rather of debility than increased power, and the condition of the nervous system is probably somewhat analogous to that which exists in delirium tremens.

Since more correct notions of the nature of the disease have prevailed, changes have taken place in the treatment. I believe, however, that, at the present time, erroneous opinions are still held which lead to an injurious line of practice, and that in many cases, especially at the onset of the symptoms, when there are great excitement and apparently increased vascular action, depressing remedies are often resorted to, which lower the patient's strength and diminish the chances of ultimate recovery. In the hands, however, of the well-informed, such treatment is not resorted to; it is replaced by remedies addressed to the nervous system. Sedatives and narcotics are the sheet-anchor. Before, however, these are administered, cathartics, more or less powerful, according to the nature of the symptoms and the condition of the patient, should be used; for almost invariably in these cases the bowels are much loaded, and much relief is obtained by evacuating their contents.

Amongst the remedies that have been used for the purpose of controlling this disease, opium occupies the first place. Almost all authors speak in high terms of its value. My experience does not in all respects agree with the opinions thus expressed. I have observed but little benefit follow its use in the severe forms of puerperal mania, and still less in other forms of mania. In the milder forms of the disease, it is, undoubtedly, of great value, and when the patient will swallow both food and medicine, and when the only indication is to procure sleep, it will often alone be sufficient to effect recovery; but there are cases in which, from the continued restlessness and obstinate refusal of the patient to take anything whatever, opium cannot be administered; and again there are other cases in which, although administered, it produces no good result, but seems rather to increase the mental excitement. In cases of this kind, the long-continued restlessness, insomnia, and absence from food, produce a state of exhaustion which, if not relieved by the introduction of nourishment into the system, and by rest, will soon terminate fatally. It is in such cases as these that we notice the great value of chloroform.

As illustrations of the line of practice I wish to recommend, and of the benefit to be derived from it, I have selected the following cases, which occurred in the Liverpool Royal Lunatic Asylum, under the conjoint care of Dr. Formby, the visiting physician to the asylum, and myself, during the period I was medical attendant to the institution :—

Case No. 1.—C. D. E., 24 years of age, of full habit and nervous temperament, was admitted into the Liverpool Royal Asylum as a patient.

Six weeks prior to admission, she was confined with a girl. She continued well for three weeks, and at the end of that period began to exhibit symptoms of a deranged state of mind. She had been of active habits, but had confined herself almost entirely to household duties. There had been no previous attack. Treatment had been adopted at her own home for a short time, and for three days before admission she had been put under restraint: during this period she had been very violent, and had refused food.

When admitted into the asylum, she laboured under alternate depression and excitement; there was an almost entire absorption in religious matters, and great irritability of temper. She was very restless and sleepless, and required constant watching, to prevent her committing violence. She refused all food, and objected to everything intended for her comfort.

There was nothing remarkable about her physical condition. She was tall and well-made. One of the mammæ showed symptoms of incipient inflammation ; the pulse was quick, and the tongue furred.

A saline aperient was ordered, and belladonna lotion to the breast.

She continued in the condition above described for four days. She refused all food, had no sleep, and was very much excited. There was, however, no heat of scalp. She was ordered effervescing draughts, with one third of a grain of morphia, every three hours ; and a blister was put to the nape of the neck. On the evening of the fifth day, in consequence of her excited condition, a powerful opiate was ordered for her, but no good result was produced. On the sixth day there was no improvement ; symptoms of exhaustion were coming on, and she was getting emaciated from want of food, which she still refused. She had had very little sleep, although she had taken the morphia regularly. She was put under chloroform, and an enema of beef-tea was administered whilst she was under its influence. The morphia was omitted. She slept for several hours after the exhibition of the chloroform, and when she awoke was much more quiet, and remained so for two days, during which she took her food. At the end of that time she again refused food, and had a partial return of her previous symptoms; and as these did not subside, she was again put under chloroform on the tenth day, and another enema of beef tea was given; the same result followed as before, only to a more marked extent. She now sensibly improved, and on the twenty-first day, eleven days after the second exhibition of the chloroform, I find the following note: "Greatly improved, eats and sleeps well, answers questions for the first time." This favourable state of affairs continued up to the forty-fourth day; on that day she became restless and excited, and chloroform was again exhibited. After that date she had no further relapse. She steadily improved, both mentally and physically, and was discharged well, after having been under treatment in the asylum nearly four months. I have lately learned that she continued well after her discharge, and has since given birth to a child, no symptoms of mania having been developed.

Case No. 2.—A. M. S., 26 years of age, of spare habit and nervous temperament, was admitted into the Asylum on ———.

A little more than three weeks prior to admission she gave birth to a boy—her fourth child. There was nothing remarkable about the labour, except that it was attended with some amount of hæmorrhage. All her previous confinements had been good, and she had always made a good recovery; but during the latter part of her last pregnancy, her health had been unsatisfactory. She became low-spirited and desponding, and fell into a low physical condition generally: she took no exercise, and suffered much from constipation of the bowels. She went on well after her confinement—except that she had but little milk—up to about ten days prior to admission—viz., about a fortnight after the birth of the child. Symptoms of a somewhat hysterical nature seem to have come on at that time, and she said she was going out of her mind. Three days before admission she became violent and excited in manner, and incoherent in speech. It was stated on her admission that she had had no regular sleep for ten days, and had taken but little food. Her general habits were said to be sedentary and temperate.

When admitted into the Asylum she was very restless, and could not be kept quiet for a moment. She was constantly talking in a very incoherent manner; she fancied she was subjected to shocks of electricity, and that she was beyond the hope of salvation. There was no peculiar physical conformation about her; she was thin, of moderate stature, and rather intelligent-looking; the pulse was rapid, and feeble. She was kept quiet, and constantly watched for three days; but as the symptoms did not mend, and she had had no sleep, she was put under the influence of chloroform for a short time. She slept but little after it, and on the following day was very restless. She was ordered a brisk cathartic. She was more quiet after the bowels had acted freely ; but the next day the restlessness and want of sleep returned. Chloroform was again exhibited at night. It produced but little effect, and the case now began to assume a serious aspect, for the patient was getting worn out, from the fact that she took but little food, and had but little sleep. In order to prevent her sinking from want of nourishment, an enema of beef-tea was administered under chloroform. She retained the injection, and slept for the first time for an hour and a-half. It was repeated on the following day under chloroform, when she slept for three hours : this was on the eleventh day after admission. She now began to take food, and to pass her motions, of a healthy character, regularly. On the twelfth day chloroform was again exhibited at night; but it produced no sleep ; and, consequently, on the following night she had ♏xxx of Battley's solution. She slept after taking the draught for five hours, and was much more quiet the next day. The medicine was repeated, but it produced no sleep, and the restlessness returned, and she again refused food. The enema of beef-tea was repeated under chloroform. For the next few days she remained tolerably quiet—slept for a few hours every night after chloroform, and took some food. On the sixteenth day she had a brisk carthartic of croton oil, which seemed to be attended with benefit.

On the eighteenth day the chloroform was omitted, and tincture of henbane was tried—administered every four hours; but it produced no sleep; and ♏xl of Battley were tried with the same result. On the twenty-first day she suddenly improved : she had been restless during the day, but in the evening she retired to bed of her own accord, and slept. From this day she began to improve in her physical condition ; but for some time there was no marked improvement mentally. She continued under treatment for upwards of seven months, and was then discharged. At that time her general health was good, the catamenia had returned, and the mind was becoming gradually restored.

I have lately learned that this patient after her discharge perfectly recovered her mental faculties.

CASE No. 3.—A female, 28 years of age, of spare habit and nervous temperament, was admitted into the Asylum on ———.

About nine weeks before admission she gave birth to a boy. No history of the confinement could be obtained ; but it was stated that for nine months previous to that event she was so ill as to be obliged to keep her bed. No account, however, was given as to what she suffered from. About a week before admission, symptoms of insanity first appeared. She became very violent at times, and threatened to throw herself from the windows of her house. She was placed under treatment, but no benefit took place. She suffered from fits of a paroxysmal character, with lucid intervals. After her admission into the Asylum she became exceedingly violent at times; she had a recurrence of fits of an epileptoid character; she was very restless, and would not answer when spoken to. She laboured under the delusion that her blood was boiling, and that she had wheels in her inside. In physical condition she was low, being much emaciated,—to such an extent even, that the pulsations of the abdominal aorta could be distinctly felt on placing the hand on the surface of the abdomen.

On the second day of her admission the fits continued, and she refused to take food; she passed a quiet night. From this date up to the twenty-eighth day, there was but little improvement. On account of her restlessness and want of sleep, she was frequently put under chloroform at night, almost always with the result of giving her a quiet night. At times she refused food, and enemata of beef-tea were administered. Morphia was tried on one or two occasions to procure rest, but without effect. She required constant watching, and was kept in the padded room. She had a great tendency to injure herself, and if an opportunity were allowed her, she would knock her limbs and head against the walls, and on two or three occasions she thrust her head through panes of glass. Frequently she would refuse food for an entire day, and on the next, eat everything placed before her. She was allowed anything she would take; but she continued up to this period much emaciated. She went on with but little alteration for two months, the chloroform being occasionally administered, and also the beef-tea enemata. She subsequently began to improve, and at the end of the seventh month she was discharged at the request of her friends, nearly well.

I have lately learned that after her discharge she perfectly recovered, and continues well.

The first case exhibits in a marked manner the beneficial influence of chloroform; the opiate treatment signally failed either to procure rest or allay the mental excitement, which increased *pari passu* with the symptoms of physical exhaustion and debility. It became urgently necessary under the circumstances at once to interfere, and check, as far as possible, the tendency to sinking which was manifest. Accordingly, chloroform was administered, and some strong beef-tea was injected into the rectum; the relief was marked and persistent for some days; and when a renewal of the symptoms occurred, a renewal of the remedy produced a renewal of the relief.

In the second case detailed, the effects produced were not so immediately striking as in the first; but it must be borne in mind that the case is an example of a class of very great severity. The most formidable symptom was the refusal of food, which was persistent to an extraordinary extent; and to so extreme a condition of exhaustion was the patient reduced, that had not some nourishment been introduced into the system at the time the injections were commenced, in all probability rapid sinking would have set in. The administration of chloroform, combined with the injections, produced an amount of sleep not previously obtained; and after the second injection, food was taken, showing that the system was beginning to rally to some extent. Chloroform soon lost its power of producing lengthened sleep in this case, and the same fate attended opiates. It, however, produced a certain effect on the nervous symptoms and moderated the mental excitement, and further, it allowed of the introduction of nourishment into the system through the medium of the enemata.

The indications for the use of chloroform were strongly marked in the third case I have reported. Something to calm the excitement, if only for a short time, was urgently called for, and opium failed to produce the effect; and, in addition, the emaciated condition of the patient rendered the introduction of food into the system absolutely necessary.

The cases I have detailed will, I think, be sufficient to point out the benefit to be derived from the use of chloroform in the severe forms of puerperal insanity; but I should by no means confine its use to such cases. I believe it is calculated to afford the best means of treating the disease when it exists in a milder form. At the *very* commencement of an attack it is likely to increase the mental excitement, and therefore its administration is not to be recommended; but when the disease has existed for a few days, it is probably the best sedative we can use. The milder forms of the affection will yield to other treatment; but it is especially in the severe forms, in which. from lengthened wakefulness, excitement, and abstinence from food, there is every prospect of sinking from exhaustion, that this remedy is so valuable. If an attempt be made to introduce food into the rectum without anæsthetic agents, the attempt will be frustrated, either by the resistance of the patient or the rejection of the enemata, but with the use of anæsthesia, no difficulty is experienced, and in no single instance have I known the rectum to put on expulsive action. The injections may be given, if necessary, two or three times a day, and the results will soon be manifested in the improved condition of the patient, and often the willingness to partake of food. In dealing with these cases, it is not as though we had to deal with patients in a sound state of mind, or suffering from organic disease. Food is required, and would be easily borne and readily digested, but the patients are unconscious of the want, and ignorant of the danger of prolonged abstinence. Nor can such cases be starved into eating. The debility which ensues aggravates the mental symptoms, the excitement often becomes greater as the case progresses, and the refusal of food more obstinate.

I have already mentioned my opinion of the value of opium in the treatment of this disease, and I have the satisfaction of knowing that the lengthened experience of Dr. Formby, the physician to the Asylum, bears out the view I entertain. It must be remembered that I now refer to severe cases, and no one will for a moment doubt that those I have detailed were of such a character. In those cases opium produced little or no benefit, although freely administered; and supposing it would act, it is only calculated to meet two of the indications required—viz., to subdue the restlessness, and promote sleep. It may be said that food will not be refused, if sleep and quiet are obtained; experience by no means bears out this view. Further, the administration of opium is calculated to check the secretions and constipate the bowels, and thus produce a condition which tends to aggravate the mental symptoms.

On the other hand, the administration of chloroform meets every indication. It procures rest and quiet, it is generally followed by a more or less lengthened sleep, its effects may be kept up for hours without, I believe, producing any injurious effect whatever, and whilst the patient is enjoying the rest which the agent affords her, she may be fed by enemata. She may even be fed by the mouth. There is no objection, under certain circumstances, to the injection of food into the stomach, the patient being placed in a chair. I performed this operation on one patient on three consecutive days. The patient was a man who for a whole week after admission refused food, not a particle of any kind passed his lips, and finding there was no possibility of making him eat, I proposed that he should be put under chloroform, and that some beef-tea should be injected into his stomach. This was accordingly done, about a pint and a half of strong beef-tea being injected through the medium of the stomach-pump. I had some fear that the fluid would be rejected, but the result was most satisfactory, every particle was retained, and on the two following days the process was repeated, on the last day without chloroform; the patient being more quiet, allowed himself to be held and have the food injected into him, although he would not swallow any of his own accord. He subsequently began to eat, and we had no further trouble with him. I believe his life was saved by this timely interference.

The injection of food, however, into the rectum, is best calculated for the cases I have alluded to, for the patient may be placed on a bed, in the recumbent posture, and thus left to sleep after the enema has been given.

The possession of an anæsthetic agent, like chloroform, to be used as I have mentioned, always affords a hope of saving the patient's life, however severe the case may be, and however great the exhaustion and debility. The action is twofold, and on this its great value rests. It calms the nervous system, and restores its tone by its sedative action and the rest it produces, and it enables the patient to receive nourishment, and thus to survive till the virulence of the disease is exhausted.

Independently of the importance of introducing nourishment into the system to prevent physical exhaustion, its beneficial influence on the disease itself must not be forgotten. That even a spare diet fol!owing a moderately good one will produce diseases of a character analogous to the one in question, has been abundantly proved, and I need scarcely allude to the remarkable instance which took place at the Penitentiary many years ago. The prisoners in that institution were, for some reason, placed from a very fair diet to an extremely moderate one, and in a short time diseases of the brain, headache, vertigo, delirium, apoplexy, and even mania, became developed. If, therefore, the withdrawal of a portion of food will produce such diseases in healthy individuals, it is but logical to infer that protracted abstinence will tend to aggravate their symptoms, and we may thus see the importance of not allowing any patients suffering from diseases of the same, or an analogous kind, to pass even a short time without receiving nourishment into the system. In the treatment of delirium tremens, our great object after procuring sleep, is to introduce nourishment, knowing well that the condition of exhaustion, which is an essential feature of the disease, and the wear and tear produced by the constant excitement and restlessness, is only to be permanently restored by giving vigour to the system; and thus it is with puerperal mania; the disease itself indicates a condition of exhaustion, and the great excitement and continuous restlessness increase the physical debility.

Chloroform has been used in cases of delirium tremens, and from the success which has attended its use its value in analogous cases might be inferred. I believe we have much to learn with reference to the use of anæsthesia in diseases depending on nervous irritability. It is by no means unfrequent to witness cases of this kind, in which the predominant symptoms are those of an excited nervous system, with great depression of the physical powers, attended by insomnia, restlessness, and refusal of food. These cases generally terminate fatally, after a more or less lengthened course, and the *post-mortem* appearances reveal an anæmic condition of the brain and body generally, attended, in some cases, by a low form of inflammation of a chronic character, and, very slight, of some portion of the viscera. Moral causes are generally the excitants of such affections, and any treatment addressed directly to the disease will be attended by little or no benefit; but by using chloroform in the manner I have described, for the purpose of calming the excitement, of producing rest, and as a means of introducing food into the system, the physical depression and tendency to sinking from exhaustion may be avoided, and we may hope that the nervous system will have time to recover from the shock it has sustained, and life may thus be saved.

---

